# Association between Alexithymia and Depression among King Khalid University Medical Students: An Analytical Cross-Sectional Study

**DOI:** 10.3390/healthcare10091703

**Published:** 2022-09-06

**Authors:** Mohammed Ahmed Aleisa, Naif Saud Abdullah, Amar Abdullah A. Alqahtani, Jaber Ahmed J Aleisa, Mohammed R. Algethami, Najim Z. Alshahrani

**Affiliations:** 1 Preventive Medicine Department, Armed Forces Hospitals Southern Region, Khamis Mushit 61961, Saudi Arabia; 2 Consultant of Preventive Medicine and Public Health, Ministry of Health, Abha 62585, Saudi Arabia; 3 College of Medicine, King Khalid University, Abha 61421, Saudi Arabia; 4 Senior Specialist of Health Services Administration, Ministry of Health, Abha 62585, Saudi Arabia; 5 Preventive Medicine and Public Health Resident, Ministry of Health, Jeddah 21577, Saudi Arabia; 6 Department of Family and Community Medicine, Faculty of Medicine, University of Jeddah, Jeddah 21589, Saudi Arabia

**Keywords:** alexithymia, depression, students, medical, psychological problem, undergraduate, Saudi Arabia

## Abstract

Alexithymia is a condition in which a person is unable to explain his/her emotions, bodily sensations, or discuss sentiments. This study aims to determine the prevalence of alexithymia and its relationships with socio-demographics and depression among medical students. A cross-sectional survey was conducted among medical students at King Khalid University (KKU), Saudi Arabia. A stratified random sampling technique was utilized for data collection using the Toronto Alexithymia Scale (TAS-20) and the Patient Health Questionnaire-9 (PHQ-9). A multiple logistic regression model was used to identify the factors associated with alexithymia. A total of 333 students participated in this study, almost two-thirds (64.6%) were from clinical years, and 51.4% were females. The prevalence of alexithymia and depression was 47.4% and 88.9%, respectively. Regression analysis showed females had a doubled risk (OR = 2.09), and students with high-income status showed less probability of having alexithymia (OR = 0.39), whereas people with chronic health problems showed a doubled risk for alexithymia (OR = 2.04). Moreover, depression was significantly associated with alexithymia (OR = 1.91). Our study revealed that the prevalence of alexithymia was high along with depression among studied samples. This raises attention towards finding measures to reduce it for the better performance of students and to avoid psychological problems in the future.

## 1. Introduction

**Figure 1:**
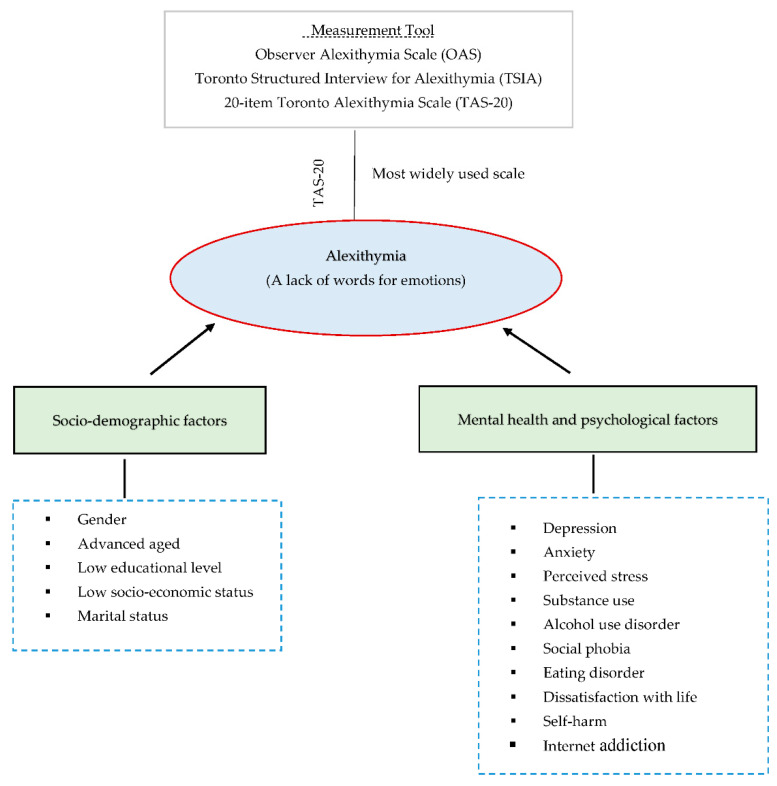
Framework indicating the social and other (such as psychological factors) determinants of alexithymia. (Note: The framework is prepared by the authors based on the literature.)

Medical education is considered one of the most difficult professions of specialized training in terms of program duration, competition, and emotional demands [1]. Students transitioning from high school to medical college may suffer from a variety of challenges, including depression, alexithymia, burnout, and anxiety [2,3]. Alexithymia was identified to be one of the most common mental disorders among students [4]. The Greek term alexithymia means “lack of words for emotions” or “inability to discover words that identify and express feelings”. It is described as a subclinical lack of emotional awareness, or more specifically, difficulties recognizing and defining feelings, as well as differentiating feelings from body sensations associated with emotional arousal [5,6]. As alexithymia is a symptom rather than a condition, it does not have any diagnostic criteria in the DSM-5 [7]. However, this symptom can worsen and lead to other mental illnesses such as depression, subjective distress, and burnout [7,8,9]. The prevalence of alexithymia varied between medical and non-medical students [10]. For example, a study carried out among Egyptian medical students showed the prevalence of alexithymia was 24.4% [11]. In addition, a study conducted by Velea et al. [12] showed the prevalence of alexithymia among Romanian medical students was 36.02%. In Arab countries, the prevalence of alexithymia was higher than in non-Arab countries, studies in Jordan and Saudi Arabia showed the prevalence among university students was 24.6% and 49%, respectively [4,13]. Medical students were found to be more prone to alexithymia due to their difficult curriculum and training [13]. According to studies, first- and fifth-year medical students had higher alexithymia scores than second-, third-, and fourth-year students [14,15]. In contrast, Morice-Ramat et al. [16] reported that there was no significant difference in alexithymia across medical students of different years of study. Nevertheless, students with alexithymia are more likely to participate in maladaptive behaviors such as suicide, substance misuse, poor academic performance, low self-efficacy, and poor self-care [7,17,18,19]. Although poor academic achievement, a lack of physical exercise, chronic illnesses, and cigarette smoking all seem to be related in some way to alexithymia, lack of family support among medical students is a crucial factor that needs to be investigated, especially its association with alexithymia [14,20]. In addition, several sociodemographic factors are associated with alexithymia among different sub-groups of the population (such as medical students, university students, or adults) including gender, advanced age, low educational level, marital status, and low socioeconomic status (see Figure 1) [4,14,21,22,23].

Additionally, medical students are susceptible to certain types of psychological distress, such as depression, anxiety, and stress, which can alter their emotional states and make it difficult or impossible for them to express and recognize their emotions [4,24,25]. According to a recent review study, depression is quite prevalent among Saudi Arabian medical students, showing the prevalence of depression varied from 30.9% to 77.6% with a mean prevalence of 51.5% [26]. Depression is a common form of mental suffering that can have a variety of clinical forms, is linked to numerous diseases, and can even lead to mortality [27]. Moreover, depression was found to be associated with alexithymia in several studies from non-Arabian countries [28,29]. Therefore, the relationship between alexithymia and depression among Saudi medical students needs to be investigated immediately because it has not been conducted yet given the extreme importance of these two issues. According to research evidence, Saudi people, similar to other Arab nations, have trouble identifying and explaining their emotions, particularly among men. Customs and cultural beliefs play a vital role in this fact, emotions are a private matter that should not be probed [13]. In Saudi Arabia, few studies have been conducted on alexithymia among medical students or university students, all of which evaluated the relationship with sociodemographic factors, students’ academic factors, and internet addiction [13,20,30]. These studies acknowledged that depression was underreported in the studies and offered recommendations for further research. Moreover, since these studies were conducted at a single institution using data from various Saudi Arabian regions, their generalization to the entire country had limitations. The southern Saudi Arabian city of Abha has cultural and sociodemographic differences (such as capital city, tourist point, climatically vulnerable, and considerably high level of mental distress among different sub-groups [31,32]) than other Saudi Arabian cities (such as Jeddah), where studies on alexithymia have been undertaken. As a result, there may be variations in the prevalence and determinants of alexithymia among medical students from Abha. In order to fill the gap in the literature and consider the regional variability, the current study sought to determine the prevalence of alexithymia and its relationships with socio-demographics and depression among medical students at King Khalid University, Abha, Saudi Arabia. The outcomes of this study will be helpful to university authorities in proposing and establishing intervention programs to diagnose and treat alexithymia.

## 2. Materials and Methods

### 2.1. Study Design and Setting

An analytical cross-sectional study was carried out among undergraduate medical students at King Khalid University, Abha, Saudi Arabia, from 11 March to 11 June 2022. A structured, anonymous, and close-ended questionnaire was used in this study. Medical students from all levels “one to twelve” of King Khalid University were invited to take part in this study. Students from other colleges, rather than medical colleges, were barred from participating, as were medical students who refused to participate.

### 2.2. Sample Size

The sample size was calculated using the single proportion equation in the Raosoft software package (Raosoft Inc., Seattle, WA, USA) [31] with a margin of error of five percent at the 95% confidence range. Based on a study published in Saudi Arabia the estimated prevalence of alexithymia was 49% [13]. The required sample size was approximately 296 students. The sample size was expanded due to a predicted lower response to an online questionnaire.

### 2.3. Sampling Technique

A stratified sampling technique was used to choose the participants. We used Google Forms and Excel sheets for data collection and entry. In addition, we sent the questionnaire via students’ university email to the participants. Prior to being asked to provide consent to participate, each participant was briefed on the study’s goal and objectives. Participants were also given the assurance that their replies to the questionnaire would remain anonymous. The participants were also told that they could withdraw from the study at any time and that it was not a requirement for their course.

### 2.4. Survey

The questionnaire (see Supplementary Materials) consisted of three parts: the first one being the demographics section, where participants were asked to identify their age, gender, smoking status, BMI, academic phase, marital status, income, and type of housing. They were then asked if they have a chronic disease and how often they engage in physical activities. The variables included in the statistical analysis were determined through the literature, and their applicability to the Saudi Arabian setting was assessed. The second part consisted of the Toronto Alexithymia Scale (TAS-20), which is used to assess the prevalence of alexithymia [32]. The TAS-20 is a self-report scale comprising 20 items that are rated using a five-point Likert scale where 1 = strongly disagree and 5 = strongly agree. The cut-off scores on the TAS-20 are ≤51 for the low end (meaning no alexithymia) and ≥61 for the high end (alexithymia). Scores between 52 and 60 indicate possible alexithymia. There is no relevant validation and adaptation research of this scale for the population of Saudi Arabia based on statistics. However, this scale has been utilized in a number of studies in Saudi Arabia and has shown that they had an acceptable level of internal consistency for the Saudi population [13,20,30,33]. We also found an acceptable level of internal consistency in this part of the questionnaire (Cronbach’s alpha: 0.77). The third part consisted of the Patient Health Questionnaire-9 (PHQ-9), which is a self-administered questionnaire used to screen for depression and assess its severity. The items were scored on a four-point scale rated from nil (not at all) to three (nearly every day) [34]. As for the PHQ-9, the overall score was obtained by totaling all discrete scores for the items that ranged from 0–27 points. The PHQ-9 scales indicated that those who had a score of 0–4 were considered normal, 5–9 was mild depression, 10–14 was moderate depression, 15–19 was moderately severe, and 20–27 was severe depression. This scale was adapted and validated in the Saudi population [35,36,37,38].

### 2.5. Ethical Consideration

All ethical considerations were assured before, during, and after conducting the study. Approval to conduct the study was obtained from the Institutional Review Board committee at the KKU (HAPO-06-B-001).

### 2.6. Statistical Analysis

The data were collected, reviewed, and then fed to the Statistical Package for Social Sciences version 21 (SPSS: An IBM Company). All statistical methods used were two-tailed with an alpha level of 0.05, considering significance if the *p*-value is less than or equal to 0.05. Descriptive analysis was conducted by prescribing frequency distribution and percentage for study variables including students’ bio-demographic data, grade, Grade Point Average (GPA), medical data, TAS-20, and PHQ-9. Alexithymia and depression prevalence and severity were graphed. Crosstabulation for factors associated with alexithymia among medical students was determined with Pearson’s chi-square test for significance and an exact probability test for small frequency distributions. A multiple logistic regression model was used to assess adjusted relationships with an odds ratio as effect size for relationships.

## 3. Results

### 3.1. Particpitants Demographic Chractristics

A total of 333 students were included. The students’ ages ranged from 18 to 27 years with a mean age of 22.3 years old. A total of 215 (64.6%) students were in their clinical years, and 171 (51.4%) were females. A total of 290 (87.1%) were single. As for BMI, 80 (24%) complained of being overweight, 48 (14.4%) had class I obesity, 22 (6.6%) had class II obesity and 6 (1.8%) had class III obesity. A total of 94 (28.2%) students were smokers, and 108 (32.4%) exercised once weekly; however, 95 (28.5%) never exercised. A total of 67 (20.1%) of students’ parents were divorced. Ninety-one students (27.3%) had a monthly income of 10,000–15,000 Saudi Riyal (SAR), while 133 (39.9%) had a monthly income exceeding SAR 15,000. A total of 114 (34.2%) had chronic health problems (Table 1).

### 3.2. Prevalence of Alexithymia and Depression 

**Figure 2:**
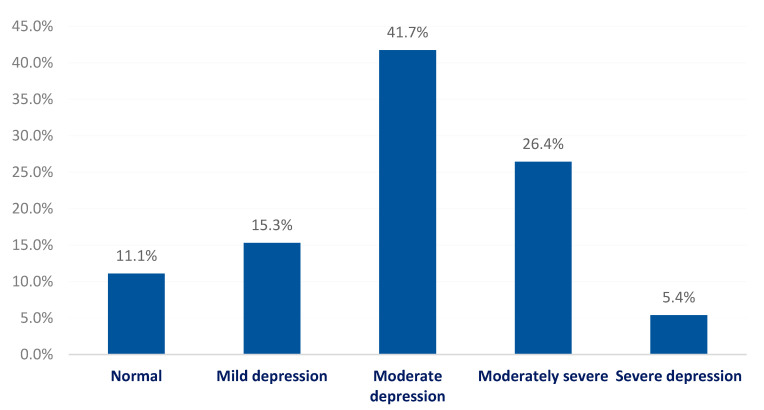
Depression severity among medical students at King Khalid University, Saudi Arabia.

A total of 158 (47.4%) medical students had alexithymia, while 135 (40.5%) had possible alexithymia and only 40 (12%) had no alexithymia. A total of 88.9% of students had depression. The prevalence of mild, moderate, moderately severe, and severe depression was 15.3%, 41.7%, 26.4%, and 5.4%, respectively (Figure 2). 

### 3.3. Relationship between Alexithymia and Depression

A total of 94.3% of students with alexithymia showed depression symptoms compared to 86.7% of students with possible alexithymia and 75% of students with no alexithymia (*p* = 0.001) (Table 2). 

### 3.4. Factors Associated with Alexithymia

A total of 52.6% of female students had alexithymia compared to 42% of male students with a recorded statistical significance (*p* = 0.048). Additionally, 56.3% of students with a family monthly income of SAR 6000–10,000 had alexithymia versus 51.9% of students with an income of more than SAR 15,000, and 35% of those with a family income of less than SAR 3000 (*p* = 0.011). Other factors were insignificantly associated with students’ alexithymia status (Table 3).

A binary multivariate logistic regression to examine the factors related to alexithymia is shown in Table 4. Among included factors, female students showed an almost doubled risk for alexithymia compared to males (OR = 2.09; 95% CI: 1.18–4.78), students of families with a high income showed less probability for alexithymia (OR = 0.39; 95% CI: 0.17–0.97), and students with chronic health problems showed doubled risk for alexithymia (OR = 2.04; 95% CI: 1.23–7.11). In addition, depression was found to be associated with alexithymia (OR = 1.91; 95% CI: 1.11–3.34).

## 4. Discussion

The current study assessed the prevalence of alexithymia among medical students in Saudi Arabia and evidenced that alexithymia was associated with sociodemographic factors (such as female, having a chronic disease, and income) and depression. As per the TAS-20 scale, the sample of this study showed a lower prevalence of alexithymia (47.3%) than other studies among medical students in other Saudi cities such as Jeddah (49%) [14], and Makkah (56.5%) [30]. However, we found a higher prevalence of alexithymia than a previous study conducted in the Kingdom of Saudi Arabia, which reported that 30.2% of university students had alexithymia and 33.8% had possible alexithymia [39]. Using the same scale, Al-Eithan et al. [35] showed that Saudi mothers of disabled children had a significantly higher degree of alexithymia. Moreover, the prevalence of alexithymia differs from Arab regions to other countries [13]. In Chinese medical students, the prevalence was found to be 34% [14], and only 6.02% [12] among Romanian medical students. The high proportion of alexithymia among these participants, especially in Saudi Arabia, might be related to a number of variables that have been linked to an increased risk of mental problems in the literature [7]. These characteristics include beliefs and prior experiences with mental health concerns, a lack of information regarding official services, social stigma, uncertainties regarding the validity of mental diseases, and the utilization of indigenous informal resources [13,40]. However, the prevalence among non-medical students such as nursing students [41], university students [4], and high school students [29] was less when we compared it with medical students. Due to the higher proportions, medical students should be aware of alexithymia’s prevalence and its consequences in their own lives as it might reflect on their performance which could influence their future as physicians [18].

Our study found significant gender differences in susceptibility to alexithymia. The current study reported female medical students had a higher risk of developing alexithymia compared to male students. This finding is consistent with the findings of several studies, including Hamaideh et al. [4] and Alharthi et al. [30], which state that females are more likely to develop emotional problems, which explains why they are more likely to develop alexithymia. In contrast, a study carried out by Zhu et al. [14] revealed that male medical students were at higher risk for alexithymia. Previous studies among young adults/general population reported that prevalence of alexithymia was higher in men than in women [21,22]. Our findings also demonstrated that students whose families have a high income were less likely to experience alexithymia. This finding is consistent with a study conducted among the Lebanese population by Obeid et al. [23], which showed that the prevalence of alexithymia decreased as income increased. Moreover, Kokkonean et al. found that alexithymia was associated with poor education and low-income status, and was common among unmarried people [21]. A previous study also found a strong association of alexithymia with increasing age among the general population [22]. However, in this study, we did not find any significant association between alexithymia and age.

In this study, we found medical students with chronic diseases were more likely to have alexithymia. Several studies revealed that chronic diseases have a high occurrence worldwide and psychological illnesses may impact patients’ capacity to manage them, as a chronic disease is an age-related problem that will be with them to the end of their lives [42,43]. Generally, alexithymia is found to be associated with mortality and morbidity, for instance, a study reported the risk of cardiovascular disease death among middle-aged Finnish men was increased by 1.2% for each 1-point increase in TAS [44].

In our study, we found the prevalence of depression among medical students was 88.9%; nearly a third of them (31.8%) had moderate to severe depression, which is relatively high when compared to several studies carried out in Saudi Arabia [26,45]. A study conducted by Alamri et al. (2020) [46] reported that 28.9% of the general population in Saudi Arabia had depressive symptoms. As per a recent study, the rate of depression among the general population of the Jazan region in Saudi Arabia was nearly 26% during the COVID-19 pandemic [47]. However, Al Rashed and his colleagues [48] found a lower prevalence of depression (8.6%) among the general population in the Al-hasa region than in other parts of Saudi Arabia. Worldwide, mental health and psychological problems of university/medical students are recognized as serious public health issues [49,50]. Medical students are more likely to develop depressive symptoms since stress level is higher in medical students compared to the general population [3,51].

This study found a statistically significant difference among medical students with severe depression as they had the highest score of alexithymia in our sample. A meta-analysis of studies using both clinical and general population samples found that alexithymia scores were moderately correlated to scores for depression severity [51]. Our regression model showed that depression is a potential risk factor for alexithymia (OR = 1.91, 95% CI: 1.11–3.34). These findings were consistent with those of previous research that showed comparatively similar results [4,28,29]. A previous study undertaken among Jordanian university students reported that alexithymia was significantly correlated with depression, anxiety, and stress [4]. Another study conducted among Lebanese adolescents found higher levels of alexithymia were significantly associated with higher depression scores [52]. These results suggested that university officials would place the mental health and well-being of medical students at the top of their priority lists. Additionally, it is strongly recommended that future research examines the relationship between alexithymia and other psychological states such as stress, anxiety, and life satisfaction of medical students in Saudi Arabia.

Literature suggests that the higher the alexithymia scores, the higher the depression scores (i.e., positive association between the presentation of alexithymia and depression) [53]. A follow-up study also revealed that following the remission of depression, alexithymia persisted to some extent [54]. This implies that alexithymia has both state-dependent (e.g., mood but also general psychopathology) and trait-dependent characteristics [53]. If strong alexithymia hinders a consistent expression of emotional changes, for example, for patients who admit to a sad mood only erratically, a phenomenological form of depression may manifest without sadness [53,55].

Alexithymia and depression are often described as similar constructs; however, according to some researchers, the subscales of alexithymia only serve to measure the pre-defined concept of depression [56]. The evidence to date indicates that depression and alexithymia are distinct but closely linked entities [56,57,58,59]. One issue is still debatable whether alexithymia is a predisposing factor (vulnerability hypothesis) or the consequence of depression (reactivity hypothesis), or whether they coexist [56,58]. However, to date, most studies have provided support for the predisposition role of alexithymia (i.e., vulnerability hypothesis). For instance, Gilanifar and Delavar [59] found that women with alexithymia had a 2.6-fold higher risk of developing depressive symptoms. 

Alexithymia is defined as a condition of poor emotional regulation that can lead to significant unpleasant feelings such as depression, anxiety, separation anxiety, and avoidance behaviors [11]. Medical students who scored highly for both alexithymia and depression in this study had more trouble with their experiences and feelings than those who did not. As a result, these students might practice harmful coping mechanisms such as smoking or risky actions such as suicidal behavior or social disengagement, leading a sedentary lifestyle, or even dropping out of college [60].

In this study, almost 40.5% of the participants had possible alexithymia, so it is necessary to keep reassessing these students to check if their scores are improving or declining over time, as some of them may be able to recover by using self-help coping methods such as spiritual coping mechanisms [13,61]. Further research should be conducted on the impact of customs and cultural beliefs on depressive symptoms and alexithymia among at-risk populations such as students, children, mothers, and the elderly. 

### Strength and Limitations of this Study

The present study has several drawbacks that should be acknowledged. First, the cross-sectional nature of this study does not allow to establish causal interferences, making it difficult to determine if alexithymia increases the incidence of depression among medical students or whether other risk factors are responsible. Secondly, the study participants (medical students) were recruited from a single medical institution; therefore, the findings cannot be generalized to the whole country. Thus, conducting a multi-centric study with an adequate sample size would enhance the generalizability of the results and could lead to significant improvements in other characteristics related to alexithymia. Finally, due to the self-report nature of the assessments utilized in this study, social desirability and reporting biases from the respondents may occur. Despite these, this study has a number of potential benefits. This was one of the first studies to assess the relationship between alexithymia and depression among medical students in Saudi Arabia. These results would add evidence to the existing body of literature, and serve as baseline data for policymakers, mental health professionals, and clinicians to assist with the development and implementation of evidence-based interventions and initiatives to improve alexithymia and mental health among medical students in Saudi Arabia. Moreover, methodological and analytical approaches represent another strength of this study. 

## 5. Conclusions

This study reported nearly half of the participants (47.3%) had alexithymia, and several sociodemographic (such as being female, having chronic health problems, and income status) and psychological factors (depression) were found to be significantly associated with alexithymia. The findings imply the necessity for thorough screening for depression and alexithymia before the commencement of the first semester in order to prevent the usually harmful repercussions that come from it. Additionally, it is highly recommended to set up training, awareness programs, and psychological interventions for students who have alexithymia so that they can accurately identify and express their emotions and feelings. Since psychological factors such as depression are linked to alexithymia, students should be routinely monitored by mental health practitioners to prevent any psychological distress. Further longitudinal, follow-up, or interventional studies that may incorporate large and diverse samples are warranted to better understand the causal interactions between alexithymia and psychological distress such as stress, anxiety, and depression.

## Figures and Tables

**Table 1:** Demographic data of medical students at King Khalid University, Saudi Arabia, 2022.

Demographic Data	N	%
Age in years		
18–20	53	15.9
21–23	202	60.7
24–27	78	23.4
Gender		
Male	162	48.6
Female	171	51.4
Academic phase		
Pre-clinical	118	35.4
Clinical	215	64.6
Marital status		
Single	290	87.1
Married	43	12.9
Body Mass Index (BMI)		
Underweight	32	9.6
Normal weight	145	43.5
Overweight	80	24
Obesity Class I	48	14.4
Obesity Class II	22	6.6
Obesity Class III	6	1.8
Smoking status		
Yes	94	28.2
No	239	71.8
How often do you take part in physical training per week?		
>3 times	47	14.1
3 times	83	24.9
1 time	108	32.4
Never	95	28.5
Parents’ status		
Married	266	79.9
Divorced	67	20.1
Family monthly income		
SAR < 3000	20	6
SAR 3000–6000	41	12.3
SAR 6000–10,000	48	14.4
SAR 10,000–15,000	91	27.3
SAR>15,000	133	39.9
Housing		
Own house	235	70.6
Rented house	59	17.7
Students’ dormitory	39	11.7
Do you have any chronic illnesses?		
Yes	114	34.2
No	219	65.8

SAR = Saudi Riyal (SAR 1 = USD 3.75).

**Table 2:** Relationship between alexithymia and depression among medical students at King Khalid University, Saudi Arabia, 2022.

TAS-20	Depression				*p*-Value
	Normal		Depression		
	N	%	N	%	
No alexithymia	10	25	30	75	0.001 *
Possible alexithymia	18	13.3	117	86.7	
Alexithymia	9	5.7	149	94.3	

*p*: Pearson X^2^ test, * *p* < 0.05 (significant).

**Table 3:** Factors associated with Alexithymia among medical students at King Khalid University, Saudi Arabia, 2022.

Factors	Alexithymia						*p*-Value
	No Alexithymia		Possible Alexithymia		Alexithymia		
	N	%	N	%	N	%	
Academic phase							
Pre-clinical	11	9.3	52	44.1	55	46.6	0.430
Clinical	29	13.5	83	38.6	103	47.9	
Age in years							
18–20	6	11.3	19	35.8	28	52.8	0.216
21–23	30	14.9	81	40.1	91	45.0	
24–27	4	5.1	35	44.9	39	50	
Gender							
Male	20	12.3	74	45.7	68	42	0.048 *
Female	20	11.7	61	35.7	90	52.6	
Body mass index (BMI)							
Underweight	4	12.5	15	46.9	13	40.6	0.908 ^$^
Normal weight	17	11.7	54	37.2	74	51	
Overweight	11	13.8	34	42.5	35	43.8	
Obese	8	10.5	32	42.1	36	47.4	
Smoking status							
Yes	14	14.9	41	43.6	39	41.5	0.334
No	26	10.9	94	39.3	119	49.8	
How often do you take part in physical training per week?							
>3 times	3	6.4	22	46.8	22	46.8	0.907 ^$^
3 times	11	13.3	34	41	38	45.8	
1 time	14	13.0	42	38.9	52	48.1	
Never	12	12.6	37	38.9	46	48.4	
Parents’ status							
Married	30	11.3	110	41.4	126	47.4	0.667
Divorced	10	14.9	25	37.3	32	47.8	
Family monthly income							
SAR < 3000	7	35.0	6	30.0	7	35	0.011 *
SAR 3000–6000	6	14.6	23	56.1	12	29.3	
SAR 6000–10,000	5	10.4	16	33.3	27	56.3	
SAR 10,000–15,000	12	13.2	36	39.6	43	47.3	
SAR >15,000	10	7.5	54	40.6	69	51.9	
Housing							
Own house	29	12.3	90	38.3	116	49.4	0.494 ^$^
Rented house	7	11.9	24	40.7	28	47.5	
Students’ dormitory	4	10.3	21	53.8	14	35.9	
Do you have any chronic illnesses?							
Yes	16	14.0	52	45.6	46	40.4	0.171
No	24	11	83	37.9	112	51.1	

*p*: Pearson X^2^ test, ^$^: Exact probability test, * *p* < 0.05 (significant).

**Table 4:** Binary multivariate logistic regression of factors associated with alexithymia among medical students at King Khalid University, Saudi Arabia, 2022.

Factors	*p*-Value	OR	95% CI	
			Lower	Upper
Female gender	0.023 *	2.09	1.18	4.78
High income (>10,000 SAR)	0.044 *	0.39	0.17	0.97
Have chronic disease	0.041 *	2.04	1.23	7.11
Depression	0.012 *	1.91	1.11	3.34

OR: Odds ratio, CI: Confidence interval, * *p* < 0.05 (significant).

## Data Availability

Not applicable.

## Supplementary Materials

The following supporting information can be downloaded at: https://www.mdpi.com/article/10.3390/healthcare10091703/s1, The study questionnaire.
