# Supplementary material for: Association between Alexithymia and Depression among King Khalid University Medical Students: An Analytical Cross-Sectional Study
Source: Healthcare (Basel). 2022 Sep 6;10(9):1703. doi: 10.3390/healthcare10091703 (PMC9498473; doi:10.3390/healthcare10091703)
Supplement: Supplementary file 1 [file healthcare-10-01703-s001.zip › healthcare-1828401-supplementary.pdf]

Association Between Alexithymia and Depression Among King Khalid University Medical Students: An Analytical Cross-Sectional Study

The study questionnaire:

Demographics:

1- **Age:**
2- Gender: male, female.
3- GPA: less than 2.5, 2.5-3.49, 3.5-4.5, more than 4.5.
4- Student Educational level: 1,2,3,4,5,6,7,8,9,19,11,12.
5- BMI: less than 18.5, 18.5 to 24.99, 25 to 29.99, 30 to 34.99, 35 to 39.99, 40 or more.
6- Marital status: single, married.
7- Smoking status: smoker, non-smoker.
8- How often do you take part in physical training: never, one time a week, three time a week, more than three time.

Toronto Alexithymia Scale (TAS-20)

The total alexithymia score is the sum of responses to all 20 items, while the score for each subscale factor is the sum of the responses to that subscale. The TAS-20 uses cutoff scoring: equal to or less than 51 = non-alexithymia, equal to or greater than 61 = alexithymia. Scores of 52 to 60 = possible alexithymia

1. I am often confused about what emotion I am feeling.

a. Strongly Disagree

b. Disagree

c. Neither Agree nor Disagree

d. Agree

e. Strongly Agree

2. It is difficult for me to find the right words for my feelings.

a. Strongly Disagree

b. Disagree

c. Neither Agree nor Disagree

d. Agree

e. Strongly Agree

3. I have physical sensations that even doctors don’t understand.

a. Strongly Disagree

b. Disagree

c. Neither Agree nor Disagree

d. Agree

e. Strongly Agree

4. I am able to describe my feelings easily.

a. Strongly Disagree

b. Disagree

c. Neither Agree nor Disagree

d. Agree

e. Strongly Agree

5. I prefer to analyze problems rather than just describe them.

a. Strongly Disagree

b. Disagree

c. Neither Agree nor Disagree

d. Agree

e. Strongly Agree

6. When I am upset. I don’t know if I am sad, frightened, or angry.

a. Strongly Disagree

b. Disagree

c. Neither Agree nor Disagree

d. Agree

e. Strongly Agree

7. I am often puzzled by sensations in my body.

a. Strongly Disagree

b. Disagree

c. Neither Agree nor Disagree

d. Agree

e. Strongly Agree

8. I prefer to just let things happen rather than to understand why they turned out that way.

a. Strongly Disagree

b. Disagree

c. Neither Agree nor Disagree

d. Agree

e. Strongly Agree

9. I have feelings that I can’t quite identify.

a. Strongly Disagree

b. Disagree

c. Neither Agree nor Disagree

d. Agree

e. Strongly Agree

10. Being in touch with emotions is essential.

a. Strongly Disagree

b. Disagree

c. Neither Agree nor Disagree

d. Agree

e. Strongly Agree

11. I find it hard to describe how I feel about people.

a. Strongly Disagree

b. Disagree

c. Neither Agree nor Disagree

d. Agree

e. Strongly Agree

12. People tell me to describe my feelings more.

a. Strongly Disagree

b. Disagree

c. Neither Agree nor Disagree

d. Agree

e. Strongly Agree

13. I don’t know what’s going on inside me.

a. Strongly Disagree

b. Disagree

c. Neither Agree nor Disagree

d. Agree

e. Strongly Agree

14. I often don’t know why I am angry.

a. Strongly Disagree

b. Disagree

c. Neither Agree nor Disagree

d. Agree

e. Strongly Agree

15. I prefer talking to people about their daily activities rather than their feelings.

a. Strongly Disagree

b. Disagree

c. Neither Agree nor Disagree

d. Agree

e. Strongly Agree

16. I prefer to watch “light” entertainment shows rather than psychological dramas.

a. Strongly Disagree

b. Disagree

c. Neither Agree nor Disagree

d. Agree

e. Strongly Agree

17. It is difficult for me to reveal my innermost feelings, even to close friends.

a. Strongly Disagree

b. Disagree

c. Neither Agree nor Disagree

d. Agree

e. Strongly Agree

18. I can feel close to someone, even in moments of silence.

a. Strongly Disagree

b. Disagree

c. Neither Agree nor Disagree

d. Agree

e. Strongly Agree

19. I find examination of my feelings useful in solving personal problems.

a. Strongly Disagree

b. Disagree

c. Neither Agree nor Disagree

d. Agree

e. Strongly Agree

20. I look for hidden meanings in movies or plays.

a. Strongly Disagree

b. Disagree

c. Neither Agree nor Disagree

d. Agree

e. Strongly Agree

The Patient Health Questionnaire (PHQ)

The total PHQ-9 score ranges from 0 to 27, with scores ≥10 being indicative of possible depression.

Over the *last 2 weeks*, how often have you been bothered by any of the following problems?	Not at all	Several days	More than half the days	Nearly every day
1. Little interest or pleasure in doing things	0	1	2	3
2. Feeling down, depressed, or hopeless	0	1	2	3
3. Trouble falling or staying asleep, or sleeping too much	0	1	2	3
4. Feeling tired or having little energy	0	1	2	3
5. Poor appetite or overeating	0	1	2	3

6. Feeling bad about yourself—or that you are a failure or have let yourself or your family down	0	1	2	3
7. Trouble concentrating on things, such as reading the newspaper or watching television	0	1	2	3
8. Moving or speaking so slowly that other people could have noticed? Or the opposite—being so fidgety or restless that you have been moving around a lot more than usual	0	1	2	3
9. Thoughts that you would be better off dead or of hurting yourself in some way	0	1	2	3
